# A Complete Cross Section Data Set for Electron Scattering by Pyridine: Modelling Electron Transport in the Energy Range 0–100 eV

**DOI:** 10.3390/ijms21186947

**Published:** 2020-09-22

**Authors:** Filipe Costa, Ali Traoré-Dubuis, Lidia Álvarez, Ana I. Lozano, Xueguang Ren, Alexander Dorn, Paulo Limão-Vieira, Francisco Blanco, Juan C. Oller, Antonio Muñoz, Adrián García-Abenza, Jimena D. Gorfinkiel, Alessandra S. Barbosa, Marcio H. F. Bettega, Peter Stokes, Ronald D. White, Darryl B. Jones, Michael J. Brunger, Gustavo García

**Affiliations:** 1 Instituto de Física Fundamental, Consejo Superior de Investigaciones Científicas, Serrano 113-bis, 28006 Madrid, Spain; filipe2626@gmail.com (F.C.); ali_traore@hotmail.fr (A.T.-D.); lid.alvarez@iff.csic.es (L.Á.); anita_ilm@iff.csic.es (A.I.L.); adrian.garcia.abenza@csic.es (A.G.-A.); 2 Laboratoire de Recherche en Ingénierie Biomédicale et Physique Médicale, Université Polytechnique Ouest Africain, Route de Ngor Almadies, Dakar 50465, Senegal; plimaovieira@fct.unl.pt; 3 Laboratório de Colisões Atómicas e Moleculares, CEFITEC, Departamento de Física, Faculdade de Ciências e Tecnologia, Universidade NOVA de Lisboa, 2829-516 Caparica, Portugal; 4 Max Planck Institute for Nuclear Physics, 69117 Heidelberg, Germany; renxueguang8@gmail.com (X.R.); dornalex@mpi-hd.mpg.de (A.D.); 5 Departamento de Estructura de la Materia Física Térmica y Electrónica e IPARCOS, Universidad Complutense de Madrid, Plaza de Ciencias 1, 28040 Madrid, Spain; pacobr@fis.ucm.es; 6 Centro de Investigaciones Energéticas Medioambientales y Tecnológicas (CIEMAT), Avenida Complutense 22, 28040 Madrid, Spain; jc.oller@ciemat.es (J.C.O.); antonio.roldan@ciemat.es (A.M.); 7 School of Physical Sciences, The Open University, Walton Hall, Milton Keynes MK7 6AA, UK; jimena.gorfinkiel@open.ac.uk; 8 Departamento de Física, Universidad Federal do Paraná, Caixa Postal 19044, Curitiba 81531-980, Paraná, Brazil; alessandra@fisica.ufpr.br (A.S.B.); bettega@fisica.ufpr.br (M.H.F.B.); 9 College of Science and Engineering, James Cook University, Townsville, QLD 4810, Australia; peter.stokes@my.jcu.edu.au (P.S.); ronald.white@jcu.edu.au (R.D.W.); 10 College of Science and Engineering, Flinders University, GPO Box 2100, Adelaide, SA 5001, Australia; darryl.jones@flinders.edu.au (D.B.J.); michael.brunger@flinders.edu.au (M.J.B.); 11 Department of Actuarial Science and Applied Statistics, Faculty of Business and Management UCSI, Kuala Lumpur 56000, Malaysia; 12 Centre for Medical Radiation Physics, University of Wollongong, Wollongong, NSW 2522, Australia

**Keywords:** electron scattering cross sections, electron transport in gases, molecular ionization

## Abstract

Electron scattering cross sections for pyridine in the energy range 0–100 eV, which we previously measured or calculated, have been critically compiled and complemented here with new measurements of electron energy loss spectra and double differential ionization cross sections. Experimental techniques employed in this study include a linear transmission apparatus and a reaction microscope system. To fulfill the transport model requirements, theoretical data have been recalculated within our independent atom model with screening corrected additivity rule and interference effects (IAM-SCAR) method for energies above 10 eV. In addition, results from the R-matrix and Schwinger multichannel with pseudopotential methods, for energies below 15 eV and 20 eV, respectively, are presented here. The reliability of this complete data set has been evaluated by comparing the simulated energy distribution of electrons transmitted through pyridine, with that observed in an electron-gas transmission experiment under magnetic confinement conditions. In addition, our representation of the angular distribution of the inelastically scattered electrons is discussed on the basis of the present double differential cross section experimental results.

## 1. Introduction

In the last few years, we have paid considerable attention, within international collaborations, to the study of electron interactions with pyridine molecules (see [1,2,3] and references therein). This is because pyridine has been considered a prototypical molecule for DNA bases, and consequently electron scattering data concerning this molecule are relevant for modelling electron damage in biomolecular systems [4]. Most of these modelling procedures [4] are based on Monte Carlo codes, which require accurate and complete [5] electron scattering cross sections in order to simulate single electron tracks into biological media. As is well known, low energy secondary electrons [6] play an important role in these models. For charged particle primary beams (electrons, positrons or ions) these secondary electrons are abundantly generated with energies below 100 eV, and therefore, electron scattering cross section data in the energy range 0–100 eV are especially needed in these types of simulations.

In this study, we present a complete cross section data set for electron scattering by pyridine, in the impact energy range (0–100 eV). These data include all the information required to simulate single electron tracks in gaseous pyridine, i.e., differential and integral elastic and inelastic (rotational, vibrational and electronic excitation, ionization) cross sections as well as electron energy loss distribution functions. The data sources are mainly the results of theoretical and experimental studies previously performed within the aforementioned international research collaboration (see Refs. [1,3]). However, here we also incorporate new electron energy loss distribution functions for the main inelastic processes (vibrational, electronic excitation and ionization), as measured with a standard electron transmission apparatus [7], and double differential ionization cross sections derived from a reaction microscope coincidence analysis [8,9,10]. The reliability of this cross section data set is then evaluated by comparing the simulated electron intensity transmitted through a well-defined molecular density of gaseous pyridine, under strong magnetic field confinement conditions, with that directly measured with an alternative electron transmission apparatus [11]. In addition, our semi-empirical formulae for the differential inelastic cross sections, derived from two different approaches, will be evaluated by comparison with those electron microscope experimental results.

The remaining sections of this paper are organized as follows. In Section 2, we present our complete set of electron scattering cross sections, which we will use for the electron transport simulations, also giving here a brief description of the corresponding data sources and expected uncertainty limits. The reliability of the present data set is discussed in Section 3, by comparing simulated and measured transmitted electron intensities for different initial electron energies. The experimental and theoretical methods used in this study are next described in Section 4. Finally, in Section 5, we will present some conclusions that have arisen through this investigation.

## 2. Results

### 2.1. Electron Scattering Measurements

As an important part of this study, we have carried out two different electron scattering experiments in order to obtain reliable energy loss distribution functions and direct measurements of the double differential (in terms of scattering angle and energy loss) cross sections (DDCS) for pyridine molecules, respectively. From the measured energy loss spectra, we will derive an average energy loss distribution function that can be used directly in the simulations. The measured DDCS will be used to check the reliability of our semi-empirical angular distribution function used for describing inelastic processes, both of these quantities are subsequently used as the input data for the electron transport simulations.

#### 2.1.1. Electron Energy Loss Spectra

**Figure 1:**
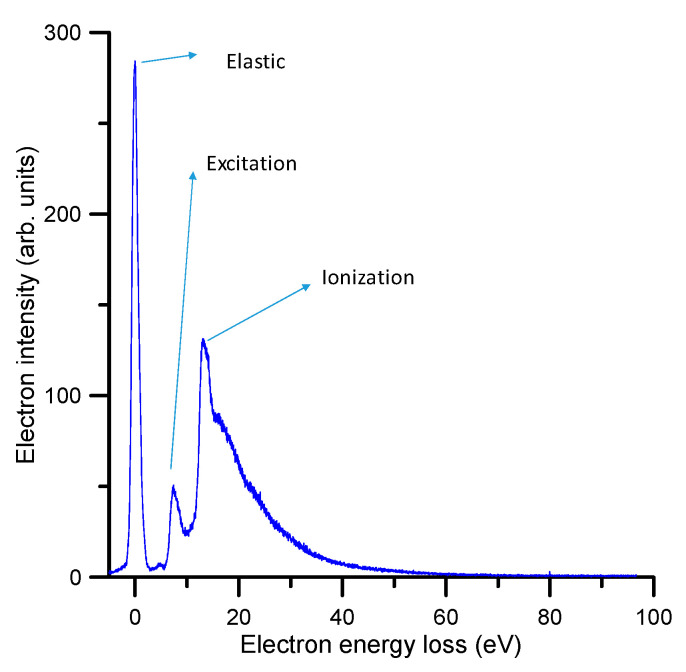
Averaged energy loss (0–100 eV) spectrum for 100 eV incident electron collisions with pyridine, as measured with our linear transmission apparatus [7].

The energy loss spectra have been measured with a standard electron transmission apparatus, which has been detailed in a previous publication [7], and is briefly described later in Section 4.1.1. Basically, it consists of a linear electron beam, which is energy analyzed with a high resolution hemispherical spectrometer, after passing through a gas cell containing pyridine at a well-defined pressure. By deflecting the beam at the exit of the gas cell, the energy loss spectra have been recorded for different incident electron energies and scattering angle intervals within the range of 0–20 deg. For impact energies above 20 eV, the obtained energy loss spectra showed a similar electron intensity distribution. A typical averaged (over the scattered electron angles from 1–20 deg) spectrum for an incident electron energy of 100 eV and for energy losses within 0 and 100 eV is shown in Figure 1.

#### 2.1.2. Double Differential Ionization Cross Sections

**Figure 2:**
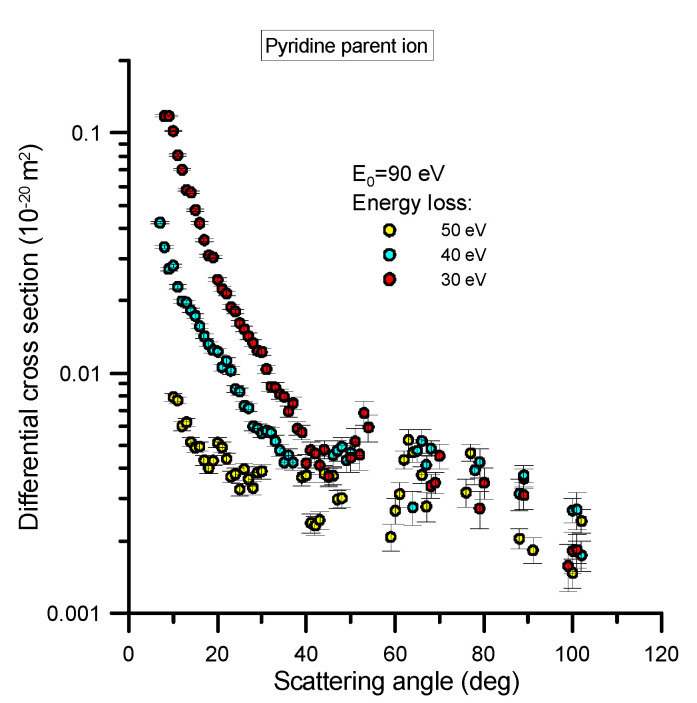
Double differential cross section for the production of the pyridine parent ion (C_5_H_5_N^+^) under 90 eV electron impact ionization where the scattered electron has different energy loss values (30, 40 and 50 eV). See also the legend in the figure.

**Figure 3:**
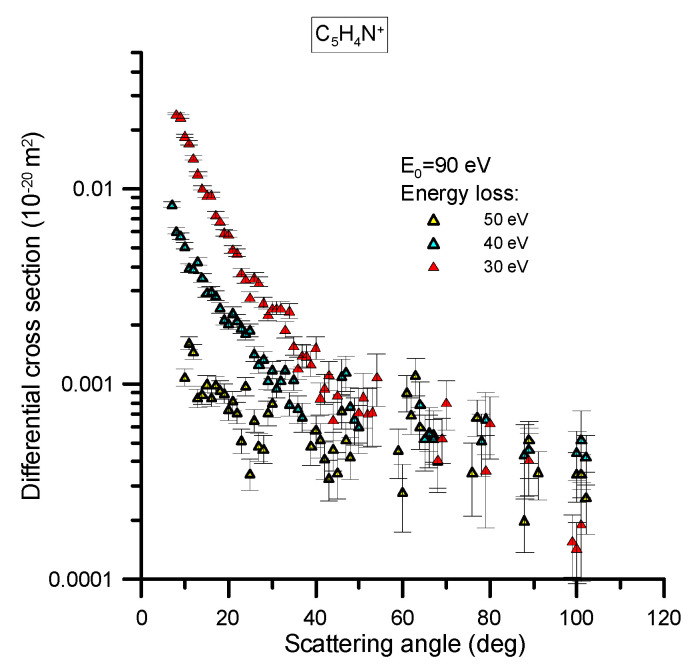
Double differential cross section for the production of the pyridine H-loss ion (C_5_H_4_N^+^), under 90 eV electron impact ionization where the scattered electron has different energy loss values (30, 40 and 50 eV). See also the legend in the figure.

The differential cross section (DCS) to ionize pyridine has been measured, with a reaction microscope, for 90 eV electron impact energy and different energy loss values, namely 50, 40 and 30 eV. The relevant experimental apparatus was designed and constructed at the MPIK institute [8] and has been recently used to investigate the behavior of different biomolecules [9,10]. The details of this experimental system have been published in these previous studies (see [9,10] and references therein), but will be briefly reiterated in Section 4.1.2. This apparatus allows for measurements in coincidence, to provide information about the angular and energy distribution of all the particles involved in the reaction (scattered electron, secondary ejected electron and recoil ion) as well as the fragmentation induced to the target species. However, for the modelling procedure we are presenting later we will only focus on the angular and energy distribution of the scattered electrons and the production of the most representative ions. Specifically, we measured this angular distribution for fixed values (30, 40 and 50 eV) of the energy transferred from the incident electron to the target molecule, and for the most probable ionization channels at the present incident energy (90 eV), which were found to be for parent ion generation (C_5_H_5_N^+^) and the H-loss fragment ion production (C_5_H_4_N^+^). The results of these particular measurements are shown in Figure 2 and Figure 3, with their statistical uncertainty limits also being shown.

The absolute scale assigned to the measured double differential cross sections (DDCS) has been derived from the normalization of the observed ionization relative to that of He atoms, where the He ionization theoretical values were calculated by Ren et al. [12] by means of the Convergent Close Coupling method. For a light atom like He, the good accuracy of this method allows one to use the calculated scattering cross sections as reference values to scale the scattering data from other species. Due to the required determination of the relative target densities of He and pyridine, which was done using the total ion yields and total ionization cross sections, we conservatively consider a 20% uncertainty associated with those absolute values. Note that this uncertainty does not actually affect upon the main goal of this study, where the relative angular distribution as a function of the energy loss will be used to check the inelastic probability distribution functions we normally employ in our modelling of electron transport [13].

As can be seen in Figure 2 and Figure 3, the DCS as a function of the scattering angle, for the ion species considered here, are very similar in shape, irrespective of the energy loss probed, although the cross sections for producing the parent ion are higher in magnitude than those for the H-loss ion fragment by a factor of ~5. For both ions, the angular distributions tend to be flat for scattering angles above 40° and for increasing energy loss values. Below 40°, however, the differential cross sections increase exponentially when the scattering angle tends to zero degrees, also showing an increment in this slope as the energy loss decreases.

### 2.2. Input Data for Modelling Electron Transport

As already mentioned, an important element of the input data for the simulations is the integral electron scattering cross sections, which give the probability for the type of interaction taking place for each collision event. Another important element is the angular distribution after the collision which is sampled, either with the differential elastic cross sections if electrons are elastically scattered or with a semi-empirical formula [13], as a function of the energy loss, for inelastically scattered electrons. A third important element for the simulations is the energy loss in the collision, which is sampled with the help of the energy loss distribution functions derived from the experimental results as described in Section 2.1.

A self-consistent and complete [5] set of data, describing all scattering processes required for modelling electron transport in the energy range 0–100 eV, is presented in the following subsections.

#### 2.2.1. Total Electron Scattering Cross Sections

**Figure 4:**
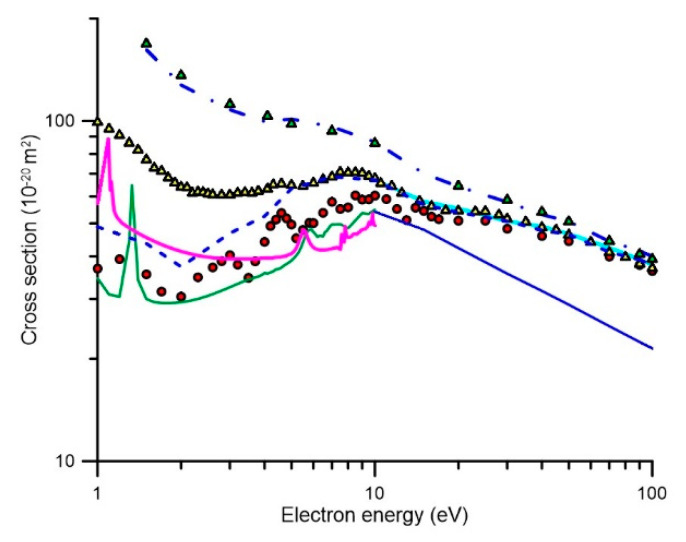
Total electron scattering and integral elastic cross sections for electron collisions with pyridine: 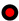, present experimental data [3]; 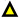, experimental data from [14]; 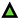, experimental data from [14] including the correction for rotational excitations into the acceptance angle of the detector (“missing angles”); **―** integral elastic cross section calculated with the SMC method; **―**, integral elastic cross section calculated with the R-matrix method; **―**, integral elastic cross section calculated with the IAM-SCAR method; **---**, our experimental data [3] including the correction for electrons elastically scattered into the “missing angles” as calculated with the IAM-SCAR differential elastic cross sections. **-.-.**; our experimental data [3] including both the corrections for elastic and rotational excitation scattered electrons into the “missing angles”.

Total electron scattering cross sections (TCS) represent the sum of the contributions from all the open scattering channels, at a given energy, and therefore they can be considered as reference values to establish a self-consistent set of scattering cross section data. In addition, their experimental uncertainty limits are customarily found to be within 5–9% using electron beam transmission experiments [3]. Two recent TCS measurements for pyridine have been published using two different techniques, namely with [3] and without [14] magnetic confinement. The corresponding results are shown in Figure 4. Above 10 eV there is very good agreement between these experimental data and also with and our IAM-SCAR calculation [1]. However, below 10 eV, the TCS data from [14] tends to be higher in magnitude, certainly well outside the stated uncertainties on each measurement, than ours. This apparent discrepancy is due to the different angular resolution used in each experiment. As discussed in [11], the angular resolution of our magnetically confined apparatus is linked to its energy resolution, which becomes poorer for lower energies, so causing the measurement to underestimate the actual TCS value. On the other hand, the angular resolution used in the transmission-beam experiment of [14] is a fixed value, given by the geometrical acceptance angle of the detector (~5 degrees). Note that as the angular resolution tends to be poorer, the “measured” TCS tends to be lower. In these circumstances, the systematic correction due to elastically scattered electrons into the “missing” angles (Δσ_el_) is negligible for the experimental data of [14] but not for [3]. Nevertheless, the latter can be corrected for by calculating the contribution of elastic scattering into the acceptance angle of the detector by using our IAM-SCAR [1] calculated elastic DCSs. As can be seen in Figure 4, the experimental data of [3], corrected by accounting for the elastic scattering into the missing angle contribution (TCS [3] +Δσ_el_), now agrees very well with those of [14] from 5 to 100 eV. Below 5 eV, however, the results from [14] again tend to be higher in magnitude than those from [3] once corrected for the “missing angle” effect. As stated above, the angular resolution used in [14] is good enough to discriminate against the contribution of electrons elastically scattered into the “missing angles”, but not totally for that of the rotational excitations. Pyridine has a significant permanent dipole moment (2.2 D [15]), thus rotational excitation cross sections, which are strongly peaked in the forward scattering direction (see Figure 3 from [3]), are dominant at the lower impact energies. As explained in [1], differential and integral rotational excitation cross sections can be reasonably easily calculated in the framework of the Born approximation. If we include the contribution of scattered electrons into the “missing angles” after the rotational excitation processes, in the correction of the experimental TCS from [3], we obtain the blue dot-dashed line also shown in Figure 4.

As may be seen in Figure 4, these latest corrected values show the same shape as that of the experimental data from [14] but tend to be higher in magnitude for the lower energies. This result suggests that in spite of the relatively good angular resolution used in the experiment of [14], it is not sufficient to fully resolve the electrons rotationally scattered in the forward direction. Note that these electrons are also not energetically resolved, given the average excitation energy for rotational processes in pyridine is about 1 meV at 300 K. In order to test this suggestion, we have added to the experimental data of [14] the contribution of the rotational excitations within the 5° acceptance angle of their detector. The result of this correction is also shown in Figure 4, as filled green triangles, where the now excellent agreement with the elastically and rotationally corrected data from [3] can be seen and considered as a confirmation for the consistency of the theoretical and experimental data involved in this analysis. As pointed out by Fabrikant [16], rotational excitation is a common source of uncertainty both for theoretical and experimental TCS determinations. A discussion about the inherent difficulties in comparing theoretical and experimental data for polar molecules, can also be found in [17]. Taking into account all these considerations, we can conclude that accurate reference values for the total electron scattering cross sections (not including rotational excitations) are the experimental data from Ref. [3] once corrected for the elastic scattering contribution to the “missing angles” (i.e., for the acceptance angle of the detector at each incident electron energy). These values, as noted earlier, are supported by the most recent experimental data of [14] for energies above 5 eV and the IAM-SCAR calculation [1] for energies above 10 eV. Considering the level of agreement between these results, we can determine a total uncertainty limit for the TCS of about 10% for energies above 5 eV. Below 5 eV, due to uncertainties introduced by the rotational excitations, we should increment this limit up to 20%.

#### 2.2.2. Integral Elastic Scattering Cross Sections

**Figure 5:**
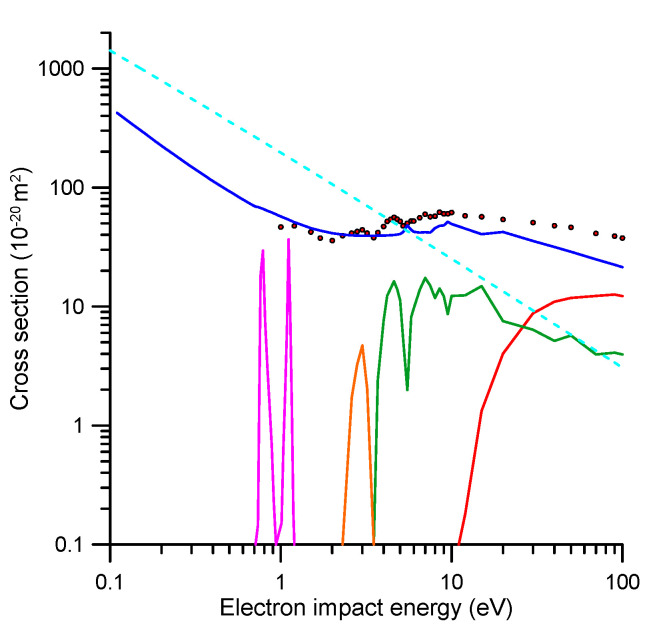
Complete set of integral electron scattering cross sections for modelling electron transport in pyridine in the energy range (0–100 eV): 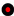, experimental data [3] including electrons elastically scattered into the “missing” angles (see text for details); 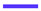, IECS based on the present R-matrix and IAM-SCAR calculations; 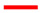, ionization; 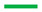, electronic excitation; 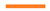, vibrational excitation; 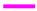, electron attachment; 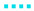, dipole-Born rotational excitation.

A detailed analysis of the elastic electron scattering cross sections for pyridine can be found in [1]. In brief, above 10 eV the IAM-SCAR calculation provides accurate integral elastic cross section (IECS) values within 10%. Below this energy, the two available “ab initio” calculations, using the R-matrix [1] and the Schwinger multichannel (SMC) with pseudopotentials [18] methods, respectively, are plotted in Figure 4. As can be seen in this figure, they agree reasonably from 5 to 10 eV and overlap well with the IAM-SCAR calculation at 10 eV. However, below 5 eV, they diverge for decreasing energies reaching a maximum discrepancy of about 50% around 1 eV. The origin of this discrepancy may be in a poor description of the background scattering in the SMC calculations, since the main goal of that study was to describe the three π* resonances. However, we should note that the SMC calculation at around 1.5 eV gives IECS values much lower (about 40%) than the corrected TCS. This is in contradiction with the fact that at these energies, the IECS equals the TCS, as the electron attachment and vibrational excitation channels are not significant at such an energy. The elastic R-matrix calculation gives values that are slightly higher in magnitude than the corrected TCS (12–17%), but in quite good agreement if we consider the uncertainty limits (about 15%) for these impact energies. We therefore consider the R-matrix calculation as the most reliable option for energies below 5 eV, as far as the IECS values are concerned. The most suitable IECS, for the whole energy range considered here (0.1–100 eV), are shown in Table 1 (at the end of Section 2) and plotted in Figure 5. Note that the R-matrix data published in [1] has been now recalculated using the UKRmol+ suite [19], both at the DCS and ICS level, including more incident energy values as required for application of the electron transport models. The IECS from [18], shown in Figure 4, were calculated with the aforementioned SMC method with pseudopotentials, and at the static-exchange-polarization (SEP) level (see [18] for details). As noted above, the origin of its discrepancy with the R-matrix and the experimental TCS data is due to a poor description of the background scattering, but for completeness we have also recalculated the SMC data by extending the impact energy range up to 20 eV. These new results are shown in Table 2 at the end of Section 2, and they will be also used in the electron transport simulation in order to investigate the influence of elastic scattering in the simulated output.

#### 2.2.3. Integral Inelastic Scattering Cross Sections

The present recommended inelastic electron scattering integral cross sections for pyridine are shown in Table 1 and plotted in Figure 5. The rotational excitation cross sections have been calculated with the Born-based procedure described in [1]. The electron attachment cross sections have been identified with and derived from the shape resonances predicted by our R-matrix calculation. Concerning the ionizing processes, the most recent total electron impact ionization cross sections (TICS) for pyridine have been measured by Jiao et al. [20] and Bull et al. [21] and show an excellent agreement between them. We therefore selected for the present ionization cross sections an average of both those sets of data, with an uncertainty limit of ~10%. TICSs calculated by applying the energy-dependent inelastic threshold procedure [22] to our IAM-SCAR calculation, are in good agreement with this experimental average to within the uncertainty limits. The ratio between the ionization and the electronic excitation cross sections, given by our IAM-SCAR calculation, provided the coefficient used to derive the latter from the total inelastic cross sections, which in turn is derived from the reference TCS values minus the integral elastic cross sections. The remaining cross sections, required to obtain the TCS values as the sum of all the considered elastic and inelastic channels, is then attributed to the vibrational excitation cross section. This procedure ensures that a self-consistent set of integral electron scattering cross section, as shown in Figure 5, is obtained.

#### 2.2.4. Angular Distribution Functions

**Figure 6:**
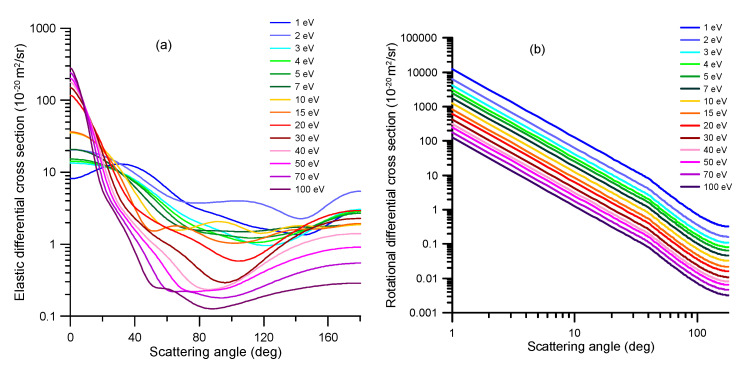
(**a**) Elastic differential cross sections, calculated with our R-matrix (1–15 eV) and IAM-SCAR (20–100 eV) methods, which are used to derive the normalized angular distribution functions for the elastic scattering processes. (**b**) Calculated dipole-Born differential cross sections used to derive the corresponding angular distribution functions for rotational excitation processes.

After any scattering event, either elastic or inelastic, for transport simulation programs, based on event-by-event Monte Carlo procedures [4], the angular distribution of the scattered electrons has to be sampled. For this purpose, in the case of the elastic processes, we simply use as the angular distribution functions our normalized elastic differential cross sections, as a function of the scattering angle, calculated with our R-matrix (for 0–10 eV) and IAM-SCAR (from 10 to 100 eV) methods. These calculated DCS, for representative impact energies, are shown in Figure 6a. Similarly, the present dipole-Born DCS calculation is used to determine the angular distribution function for the rotational excitation processes (see Figure 6b). As this figure shows, dipole-Born DCS calculations yield identically shaped curves that simply decrease in magnitude as 1/E.

For the other inelastic processes, we normally employ the following semi-empirical formula, which provides the inelastic angular distribution as a function of the corresponding elastic DCS and the energy transferred in the scattering event (see [13] for details):

(1) $$\frac{d^{2}\sigma \left(E\right)}{d\Omega \Delta E}\propto {\left(\frac{d\sigma \left(E\right)}{d\Omega }\right)}_{el}^{1-\Delta E/E}{}^{}$$

**Figure 7:**
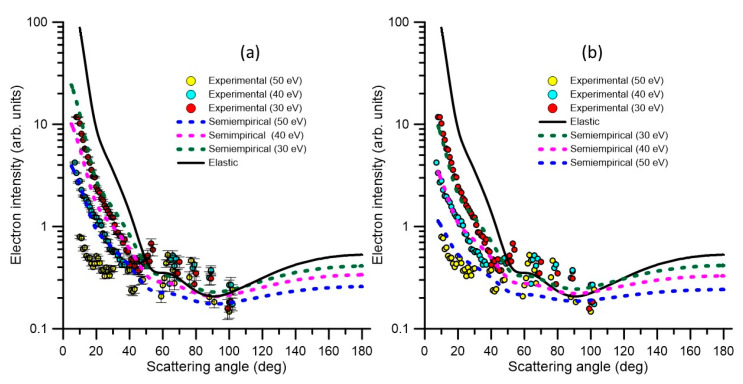
Comparison between the angular distributions given by the semi-empirical formulae (Equation (1) or (2)) for energy losses of 0 (elastic + rotational), 30, 40, and 50 eV, and those measured with the present reaction microscope for a 90 eV electron incident energy. (**a**) Using our previous semi-empirical formula (Equation (1)) and (**b**) using the present improved formula (Equation (2)) which is used to extrapolate to other energy losses.

However, from the present measured double differential ionization cross sections, for 90 eV impact energies, this formula is found to be not very accurate for relatively high values of the energy transferred in the collision process. By correlating in these cases the observed angular distribution with the calculated elastic DCS, we derived the following semi-empirical formula:

(2) $$\frac{d^{2}\sigma \left(E\right)}{d\Omega \Delta E}\propto {\left(\frac{d\sigma \left(E\right)}{d\Omega }\right)}_{el}^{{\left(1-\Delta E/E\right)}^{k}}{}^{\;\;\;\;\;\;\;\;\;\;\;\;\;\;\;\;\;\;\;\;\;\;\;\;\;\;\;\;\;\;}$$

where the best fit to the experimental data has been found for *k* = 1.3. As shown in Figure 7, this new formula reproduces the results from the previous one for low energy transfer values and fits reasonably well the observed angular distribution for the relatively high values of the incident electron energy loss. We have therefore adopted this new semi-empirical formula for our modelling purposes.

#### 2.2.5. Energy Loss Distribution Functions

**Figure 8:**
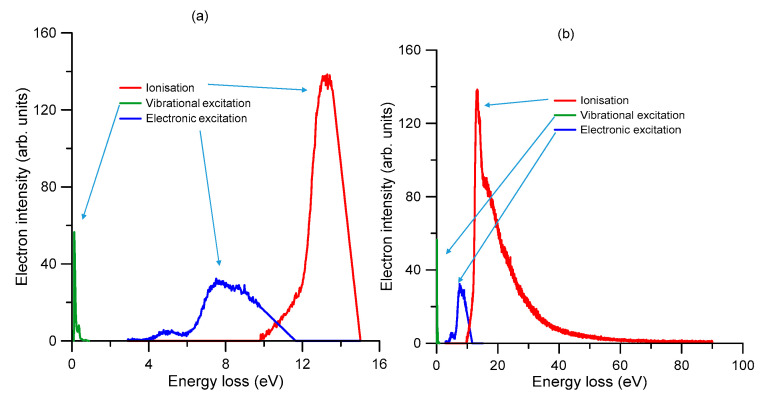
Angle-averaged energy loss distribution functions for (**a**) 15 eV and (**b**) 90 eV incident electron energies, as used in the present simulations. See also the legend in the figure to define the various open scattering processes.

The energy loss distribution functions used for the present simulation have been derived from angular averaged experimental energy loss spectra, as described in Section 2.1.1. Figure 8 shows the energy loss distribution functions used for (a) 15 eV and (b) 90 eV incident electron energies, showing the partial distributions for three of the different inelastic channels considered in our simulation, i.e., vibrational excitation, electronic-state excitation and ionization. For the rotational excitations, a fixed energy loss of 0.00109 eV (averaged rotational excitation energy at 300 K) has been assumed. For the DEA processes, the incident electrons disappear from the simulation, transferring all their kinetic energy to the medium. On the other hand, for elastic processes, only kinetic energy is transferred from the incident electron to the target molecule, with its magnitude being determined for each collision energy by their relative mass ratio. Below 0.1 eV, electrons are culled from the simulation.

## 3. Discussion

**Figure 9:**
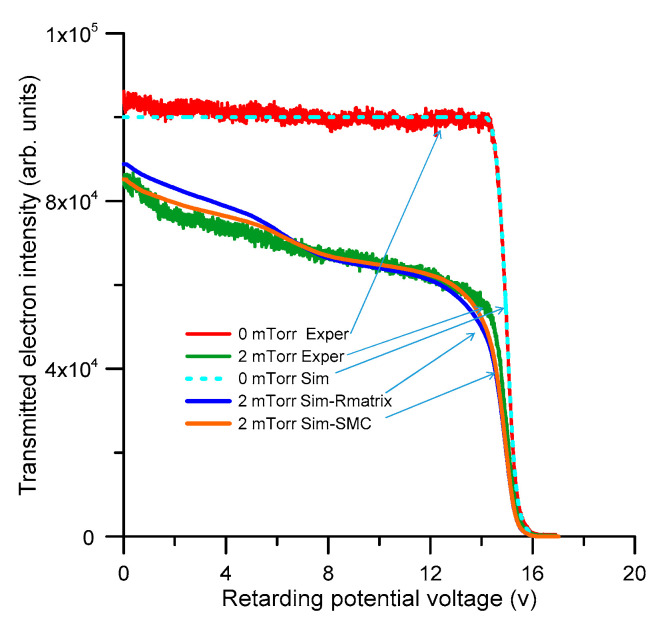
Transmitted electron intensity distribution (i.e., the intensity of the electrons with axial kinetic energy above the retarding potential barrier) through 0 and 2 mTorr of pyridine, respectively, for a 15 eV incident electron energy beam.

The complete set of consistent cross section data, presented in the previous section, is now used to simulate the electron transport through a gas cell containing pyridine, under intense magnetic field confinement conditions. Details on the experimental conditions and procedures will be given in the next section. Basically, a linear electron beam, generated by an emitting filament, is accelerated along the axis of a cylindrical chamber where it is guided by a uniform axial magnetic field and into that chamber a constant well-defined pressure of gaseous pyridine is introduced through a leak valve. The applied magnetic field is sufficiently intense (around 0.1 T) to confine the gyro-motion of the electron beam into a gyro-radius of a few microns, which is therefore negligible in comparison with the electron beam radius that is defined by the entrance and exit apertures of the chamber (each being 1.5 mm in diameter). Under these conditions, in any collision event the scattering angle is converted into a decrement of the parallel (axial) component of the velocity, which in turn represents a kinetic energy loss in the parallel direction, which can be measured with a potential barrier applied in front of an electron multiplier detector, i.e., with a retarding potential analyzer (RPA). The measured electron intensity distribution of the parallel kinetic energy, for a 15 eV electron beam generated in these experimental conditions, is plotted in Figure 9 together with the corresponding intensity distribution when a gas pressure of 2 mTorr of pyridine is introduced into the scattering chamber. The corresponding electron intensity distributions simulated with our Low Energy Particle Track Simulation (LEPTS) procedure [4,13] are also plotted in Figure 9. LEPTS is an event by event Monte Carlo simulation code which, according to the above integral cross section data set and the angular and energy-loss distribution functions, is sampling when a collision event is taking place and the type of interaction corresponding to this event as well as the energy transferred to the molecular target, the secondary particles generated and the scattering angle corresponding to both the primary and secondary electrons. Under the magnetic confinement conditions used in this experiment, the scattering angle distribution function is crucial to define the energy distribution along the magnetic axis. We can say that this particular configuration is especially suitable to evaluate the efficacy of the input DCS data used in the simulation.

As shown in Figure 9 for a 15 eV incident electron energy, considering the strong dependence of the simulated electron intensity distribution on the elastic and inelastic DCS values used as the input information, the agreement between the experiment and the simulation is fairly good, but not perfect. In the case of *p*-Benzoquinone [23], we recently obtained an excellent agreement between the simulated and experimental electron intensity distribution under similar conditions to the present. Both molecules have a similar molecular structure but the main difference is that pyridine has an important permanent dipole moment while *p*-Benzoquinone is non-polar. The limitations of the Born-type procedures used to account for the dipole interactions in our electron scattering cross section calculations have been pointed out in [16,17], and could justify, at least in part, these observed discrepancies. Note that the simulation is overestimating the effect of the angular dependence in the scattering, so slightly increasing the electron energy loss, which in turn increments the intensity of the low energy electrons, in comparison with the experimental result. From a closer inspection of Figure 9, we observe that the intensity distribution given by the simulation based on the SMC calculation better fits the experimental result than that based on the R-matrix data. This is an apparent contradiction with the fact that the IECS derived from the SMC calculation does not match very well the experimental TCS, when the “missing angle” limitation is considered. However, as already mentioned, the magnetically confined conditions of this experiment cause the simulated transmitted intensity to be very sensitive to the input DCS values. Although the IECSs for 10–15 eV given by the SMC method are higher in magnitude than those of the R-matrix, their corresponding DCSs are more peaked in the forward direction, thus producing less important energy loss than that derived by the R-matrix method. As a consequence of this, the SMC calculation gives a transmitted electron intensity distribution closer to the experimental one.

**Figure 10:**
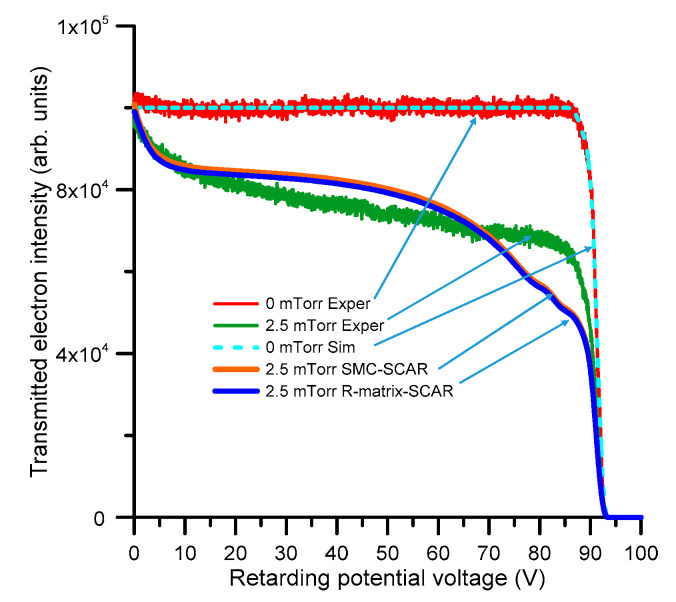
Transmitted electron intensity distribution (i.e., the intensity of the electrons with axial kinetic energy above the retarding potential barrier) through 0 and 2.5 mTorr of pyridine, respectively, but now for a 90 eV incident electron energy beam.

It is also interesting to see what happens at higher impact energies, where the ideal magnetic confinement conditions assumed in the simulation might not apply. These results are shown in Figure 10, where the experimental electron intensity transmitted through the gas cell containing 0 and 2.5 mTorr of pyridine, respectively, are plotted together with the corresponding simulation for an incident electron energy of 90 eV. As shown in this figure, there is a clear disagreement between the observed and simulated transmitted electron intensity distributions in this case. The SMC and R-matrix results do agree well with each other, but this is normal since for 90 eV the scattering data used for the LEPTS program are largely those calculated with the IAM-SCAR procedure, which is common for both sets of data for incident electron energies above 20 eV.

The observed differences between the measurement and simulation in Figure 10, seem to indicate that the angular distribution function derived from the calculated elastic DCS, as used in the simulation, tends to favour the larger scattering angles more than what the measured transmitted intensity distribution suggests is actually the case. The IAM-SCAR elastic DCS at 90 eV is supposed to be accurate to within 10%, as deduced from comparisons with accurate DCS measurements for different aromatic molecules and other halogenated compounds [24,25]. We have not found any experimental DCS data for pyridine at around 100 eV, but the recent calculation from Gholami et al. [26] shows a reasonable agreement with the present calculation at 100 eV. Assuming that the calculated IAM-SCAR DCS at 90 eV are correct, a possible reason for the discrepancy between the experimental and simulated electron transport results in Figure 10 may be lack of effectiveness of the magnetic field to confining the electron beam at that relatively high energy. Increasing the magnetic field intensity along the scattering chamber, to explicitly test that hypothesis, would require some technical modifications in the current experimental setup, which will be the subject for further investigations. Candidates for future transmission studies are the molecules benzene and nitrobenzene, which are prototype cyclic molecules without and with a permanent dipole moment, respectively.

As mentioned in the introduction of this article, a further application of the present modelling procedure would be the evaluation of radiation-induced molecular damage in biological media. Since early experiments showing that low energy electrons can efficiently damage DNA [27], a great effort has been made to obtain electron interaction data for DNA bases in the condensed phase [28]. In these conditions, below 20 eV, electron scattering data for single molecules are affected by the structural arrangement of the condensed medium. We have introduced a geometrical procedure to partially account for this effect, which is able to provide results on the energy deposition and stopping power in good agreement with the experiment [29]. However, a detailed description of the induced molecular damage would require accurate experimental fragmentation patterns for condensed molecules [30]. This is one of our current lines of research [30], which may lead to a realistic comparison between the simulated radiation-induced damage to DNA and the experimental results with condensed molecules.

## 4. Materials and Methods

### 4.1. Experimental Systems

The electron scattering cross sections and energy loss spectra presented in Section 2 have been measured with two different experimental arrangements. An additional experimental set up has also been utilized to compare the observed electron transmission intensity distribution with that simulated with the aforementioned Monte Carlo procedure based on the present scattering data. These three experimental setups are now briefly described in the following subsections.

#### 4.1.1. Linear Transmission-Beam Electrostatic Spectrometer

The experimental configuration used for energy loss measurements at intermediate and high energies is based on that used in previous studies [2,7,31], but presents some recent modifications in order to adapt the system to the requirements of the present measurements. The electron beam is generated by a negatively biased (V_C_) thoriated tungsten hairpin filament, being focused and then deflected towards the scattering chamber (SC). Typical electron currents were 10^−7^ A with an energy spread of about 600 meV. The SC consists of a 50 × 50 × 50 mm^3^ metallic (Dural) cube, defined by two 2 mm diameter apertures, which are separated by a 50 mm length (L). At the entrance of the SC, two further 1.5 mm collimators ensure that the electron beam diameter at this entrance is less than the collimator diameter so preventing possible gas focusing effects. Electrons emerging from the SC pass through a deflecting plate and lens tube system, and then are energy-analyzed with a hemispherical electrostatic spectrometer. Transmitted electrons are finally detected with a two-stage microchannel plate (MCP) operating in a single counting mode. The temperature (T) is derived from $T=\sqrt{T_{C}T_{m}}$ where *T*_C_ and *T*_m_ are the temperature of the scattering chamber, as measured with a thermocouple, and the Baratron gauge operating temperature. The accuracy of our pressure measurements is assumed to be better than 1%, as stated by the Baratron manufacturer (MKS Instruments, Inc., Andover, MA 01810, United States).

One relevant feature of this apparatus is the angular acceptance of the hemispherical spectrometer, which is used as the energy analyzer of the transmitted electrons. The 1.5 mm diameter entrance aperture of the analyzer is placed at 400 mm from the center of the SC, so that the solid angle subtended by the detector is of the order 10^−5^ sr, leading to a practical acceptance angle of about 0.25°.

#### 4.1.2. Double Electron-Ion Imaging Spectrometer (Reaction Microscope)

Double differential (angle and energy transfer) cross sections for the production of two different ionic species (C_5_H_5_N^+^ and C_5_H_4_N^+^, respectively) have been measured with a reaction microscope, specifically designed for electron-impact experiments. The principles and technical details of reaction microscopes can be found in previous publications (see [8] and references therein), so we only briefly summarize here the most representative aspects of the applied technique. The electron beam is generated by a pulsed ultraviolet laser (266 nm wavelength) focused on a tantalum photocathode, producing photo-electron pulses of about 0.5 ns duration and 0.5 eV energy spread. That electron beam is focused (1mm diameter) onto the centre of the reaction chamber, where it crosses a supersonic molecular jet. This is produced by expanding a mixture of 1 bar helium and 150 mbar pyridine through a 30 µm nozzle and forming a geometrically well-defined beam by two apertures. Uniform electric and magnetic fields define the trajectories of all the involved particles, i.e., the primary electrons, secondary electrons and the fragment ions, which are detected in coincidence by the corresponding 2-dimensional position sensitive charged particle detectors. More details on this technique can be found in a recent publication devoted to the study of electron-THF ionizing collisions [32].

#### 4.1.3. Magnetically Confined Electron Transmission Spectrometer

The electron intensity distributions for an electron beam passing through a well-defined gas density of pyridine in magnetically confined conditions, have been measured with the experimental configuration described by Lozano et al. [11]. Basically it consists of five differentially pumped vacuum areas: the electron beam generation chamber, the gas trap container, an interface compartment, the scattering chamber, and the electron analyser-detector region. Each area is surrounded by independent coils in order to generate respective axial magnetic fields to confine the electron beam along the transmission axis. As described in [11], the gas (nitrogen) trap is required to reduce the energy spread of the incident electron beam, in order to accurately measure the total electron scattering cross sections, especially around any possible low-energy electron resonances. In the present experiment, narrowing the energy distribution of the electron beam is not required since its natural energy distribution (typically 0.4–0.5 eV) is used as the initial electron energy distribution for the simulation. Note that it is even more challenging to correctly reproduce the transmitted intensity distribution for a broader energy resolution. Except for this slight difference, all the relevant experimental details and procedures of this experimental system are as described in [11].

### 4.2. Theoretical Calculations

As mentioned earlier, we have used three different methods to recalculate and adapt the available theoretical data, to the requirements of the present simulation, depending on the considered energy range. For the lower energies (0–20 eV), the Schwinger multichannel (SMC) method implemented with pseudopotentials and the R-matrix procedure have been alternately used. For higher energies (20–100 eV), the independent atom model with screening corrected additivity rule (IAM-SCAR) and interference term method has been used. These three methods have been extensively used by some of the respective co-authors of this article, and details on those calculation methods can be found in previous publications from Costa et al. [33], Mašín et al. [19] and Blanco and García [34]. In order to include dipole interactions, within the SMC and R-matrix schemes, the so-called “Born closure” procedure is commonly used [35]. However, both R-matrix and SMC data sets used in this study do not include the Born correction. Otherwise, dipole rotational excitations within the framework of the First Born Approximation have been calculated to complement the present theoretical data (see [1] and references therein). The limitations of the Born approximation to properly account for dipole interactions in electron scattering cross section calculations have been pointed out by Fabrikant [16] and discussed by us in a recent publication [17].

## 5. Conclusions

A self-consistent and complete set of differential and integral electron scattering cross sections for pyridine, based on our previous measurements and calculations, has been presented for modelling purposes, for the electron impact energies 0.1–100 eV. Our low-energy electron scattering cross sections have been recalculated by extending the incident energy range up to 20 eV, and including more incident energies into the differential elastic cross section computations. From the differential elastic cross sections calculated within these methods, and complemented with our IAM-SCAR calculations for energies above 20 eV, angular distribution functions have been derived for our modelling purposes. In order to derive the complementary energy-loss distribution functions, averaged energy-loss spectra have been measured in this energy range by using an electron transmission spectrometer. In addition, double differential ionization cross sections have been measured with a reaction microscope for 90 eV electron incident energy, and different energy transfer values (30, 40 and 50 eV), to produce results for the parent ion (C_5_H_5_N^+^) and the proton-loss ion (C_5_H_5_N^+^). Those latter experimental results have been used to check the validity of the semi-empirical formula we normally use to model the angular distribution of electrons inelastically scattered from molecules [13]. As a result of that comparison, our previous semi-empirical formula has been improved to account for higher energy-loss values. The energy distributions of electrons transmitted through pyridine for 15 eV and 90 eV incident electron energies, respectively, have been simulated with an event by event Monte Carlo procedure using the present cross section data and the angular and energy loss distribution functions as input information. Results from these simulations are compared with experimental transmission spectra, obtained with a magnetically confined transmission-beam apparatus. For 15 eV we found a reasonable level of agreement between the experimental and simulated spectra, so confirming the reliability of the present cross section data set. Slight discrepancies here did, however, suggest that the Born model needs to be improved to account for dipole interactions, and we also found that the SMC calculation provided a more reliable elastic angular distribution than the R-matrix procedure, although the latter gives ICS values in better agreement with the experimental data. At 90 eV, the observed disagreement between the simulated and measured transmitted experiments indicated some failure of the magnetic confinement conditions, for the higher energies, suggesting that an upgrade of the present experimental configuration, to allow for more intense magnetic fields, might be in order.

## Figures and Tables

**Table 1:** Complete set of integral electron scattering cross sections for pyridine, in the energy range (0.1–100 eV), in SI units (10^−20^ m^2^).

E(eV)	IECS R-Matrix [1]	Electron Attachment	Electronic Excitation	Vibrational Excitation	Ionization	Rotational Excitation
0.11	423					1374
0.135	340					1145
0.16	284.1					984.4
0.185	244.1					864.9
0.21	214.1					772.5
0.235	190.8					698.8
0.26	172.3					638.6
0.285	157.2					588.5
0.31	144.8					546
0.335	134.3					509.5
0.36	125.3					477.8
0.385	117.6					450.1
0.41	110.9					425.6
0.435	105.1					403.7
0.46	99.89					384.1
0.485	95.27					366.4
0.51	91.14					350.3
0.535	87.42					335.7
0.56	84.05					322.3
0.585	80.99					310
0.61	78.18					298.7
0.635	75.61					288.2
0.66	73.23					278.4
0.685	71.06					269.3
0.71	69.23	<0.1				260.9
0.735	68.64	0.1459				252.9
0.76	67.39	18.25				245.5
0.785	66.19	29.47				238.5
0.81	65.01	7.442				232
0.835	63.87	3.107				225.8
0.86	62.77	1.479				219.9
0.885	61.7	0.6735				214.4
0.91	60.67	0.2315				209.1
0.935	59.66	<0.1				204.1
0.96	58.61					199.4
0.985	57.72					194.9
1	57.21					192.2
1.01	56.88	0.1512				190.6
1.03	56.02	0.6431				186.4
1.06	55.2	1.899				182.5
1.08	54.4	6.533				178.8
1.11	53.65	36.67				175.2
1.13	52.93	9.549				171.7
1.16	52.25	1.578				168.4
1.18	51.59	0.184				165.3
1.2	51.09	<0.1				163.4
1.5	45.51					133.9
1.7	43.42					119.8
2	41.37			<0.1		103.6
2.3	40.23			1.73		91.51
2.6	39.64			3.26		82.04
2.8	39.44			4.71		76.8
3	39.33			2.05		72.22
3.2	39.31			<0.1		68.18
3.5	39.34		<0.1			62.95
3.7	39.4		2.47			59.91
4	39.51		7.53			55.89
4.2	39.6		12.32			53.51
4.4	39.73		14.27			51.33
4.6	39.9		16.33			49.34
4.8	40.18		14.03			47.5
5	40.77		11.25			45.81
5.2	42.28		5.26			44.23
5.5	48.05		1.99			42.08
5.8	44.2		8.13			40.13
6	42.52		9.65			38.94
6.5	41.8		13.83			36.26
7	42.21		17.40			33.94
7.5	41.79		15.01			31.91
8	45.49		11.82			30.13
8.5	47.75		14.18			28.55
9	47.94		12.21			27.13
9.5	51.54		8.65			25.85
10	49.32		12.27		<0.1	24.7
12	45.19		12.43		0.18	20.99
15	40.6		14.83		1.329	17.21
20	42.28		7.548		4.012	13.32
30	35.56		6.392		8.778	9.277
40	31.64		5.13		10.97	7.179
50	28.84		5.71		11.81	5.884
70	24.92		3.96		12.27	4.36
90	22.4		4.10		12.59	3.485
100	21.42		3.96		12.26	3.173

**Table 2:** Integral elastic electron scattering cross sections for pyridine in the energy range 0.1–20 eV, in SI units (10^−20^ m^2^), as calculated with the SMC method (see text for details).

E (eV)	IECS (10^−20^ m^2^)	E (eV)	IECS (10^−20^ m^2^)
0.1	172.4	4.1	35.58
0.2	97.4	4.2	35.86
0.3	65.32	4.3	36.14
0.4	50.62	4.4	36.62
0.5	42.81	4.5	36.73
0.6	37.78	4.6	37.05
0.7	34.31	4.7	37.58
0.8	32.47	4.8	37.96
0.9	81.96	4.9	38.38
1	34.43	5	38.87
1.1	30.87	5.1	39.47
1.2	30.44	5.2	40.2
1.3	44.29	5.3	41.04
1.33	64.7	5.4	42.3
1.4	34.02	5.5	43.94
1.5	29.95	5.6	45.72
1.6	29.33	5.7	47.19
1.7	29.17	5.8	47.82
1.8	29.14	5.9	47.98
1.9	29.2	6	47.16
2	29.31	6.1	46.48
2.1	29.49	6.2	45.63
2.2	29.72	6.3	45.94
2.3	29.98	6.4	45.94
2.4	30.29	6.5	45.76
2.5	30.62	6.6	47.07
2.6	30.97	6.7	47.49
2.7	31.34	6.8	48.26
2.8	31.71	6.9	49.14
2.9	32.08	7	49.62
3	32.46	7.5	49.71
3.1	32.83	8	48.35
3.2	33.19	9	53.61
3.3	33.54	9.5	53.28
3.4	33.89	10	54.85
3.5	34.19	11	56.67
3.6	34.39	12	52.58
3.7	34.64	15	43.31
3.8	34.92	16	46.12
3.9	35.2	18	39.85
4	35.74	20	36.35

## Abbreviations

IAM-SCAR	Independent atom model with screening corrected additivity rule and interference effects
SMC	Schwinger multichannel
DCS	Differential cross section
DDCS	Double differential cross section
MPIK	Max-Plank institute for nuclear physics
TCS	Total cross section
ICS	Integral cross section
IECS	Integral elastic cross section
SEP	Static-exchange-polarization
TICS	Total ionization cross section
DEA	Dissociative electron attachment
LEPTS	Low energy particle track simulation
EEL	Electron energy loss
SC	Scattering chamber
MCP	Micro channel plate
PC	Personal computer
RPA	Retarding potential analyzer
THF	Tetrahydrofuran
